# Hypergravity upregulates renal inducible nitric oxide synthase expression and nitric oxide production

**DOI:** 10.18632/oncotarget.9253

**Published:** 2016-05-09

**Authors:** Gun Yoon, Choong Sik Oh, Hyun-Soo Kim

**Affiliations:** ^1^ Department of Obstetrics and Gynecology, Pusan National University Yangsan Hospital, Pusan National University School of Medicine, Yangsan-si, Gyeongsangnam-do, Republic of Korea; ^2^ Aerospace Medicine Research Center, Republic of Korea Air Force Aerospace Medical Center, Cheongju-si, Chungcheongbuk-do, Republic of Korea; ^3^ Department of Pathology, Severance Hospital, Yonsei University College of Medicine, Seoul, Republic of Korea

**Keywords:** inducible nitric oxide synthase, nitric oxide, hypergravity, kidney, Pathology Section

## Abstract

Exposure to hypergravity severely decreases renal blood flow, potentially causing renal dysfunction. Nitric oxide (NO), which is endogenously synthesized by inducible NO synthase (iNOS), plays an important role in the regulation of renal function. The purpose of this study was to examine the effect of hypergravity exposure on the production of NO in kidneys. To determine whether hypergravity induces renal hypoxia and alters renal iNOS expression and NO production, mice were exposed to short-term hypergravity at +3Gz for 1 h. The time course of iNOS mRNA expression, hypoxia-inducible factor (HIF)-1α expression, and NO production was examined. Renal HIF-1α levels were significantly elevated immediately after centrifugation, and this increase was sustained for 3 h post-exposure. iNOS mRNA levels were also significantly increased immediately after exposure and were maintained during the reoxygenation period. Immunohistochemical staining for iNOS revealed that the cortical tubular epithelium exhibited moderate to strong cytoplasmic iNOS immunoreactivity immediately after hypergravity exposure and during the reoxygenation period. The time course of NO production was similar to that of iNOS expression. Our results suggest that both hypoxia and reoxygenation might be involved in the upregulation of HIF-1α in the kidneys of mice exposed to hypergravity. Significant increases in renocortical iNOS expression immediately after centrifugation and during the reoxygenation period suggest that iNOS expression induced by hypergravity exposure might play a protective role against hypoxia/reoxygenation injury in the renal cortex. Further investigations are necessary to clarify the role of iNOS and NO in kidneys exposed to hypergravity.

## INTRODUCTION

The development of a new generation of combat aircrafts with extended flight capabilities raises the problem of protection of the crew members against visceral organ diseases due to sustained gravitational (G) force exposure [1]. Exposure to high G force is conclusively known to be harmful to the human body during aviation activities [2]. With rapid developments in aviation technology, pilots are frequently exposed to hypergravity. Modern, high-performance aircrafts involve sustained and repeated exposure of the crew to high G force that may exceed the limit of physiological tolerance, possibly resulting in pilot incapacitation and subsequent loss of life. Exposure to hypergravity, particularly when it occurs repeatedly, may cause cumulative, adverse stress responses in the body [3].

Structural and functional alterations in the visceral organs are the preferred targets of the pathophysiological effects of hypergravity. The G force is a unique stress that principally results in impaired visceral blood flow when the inertial vector is in the head-to-foot direction (+Gz) [1, 2, 4]. Changes in the visceral blood flow may be the result of some combination of +Gz-induced cardiovascular reflex responses and emotional stress that causes sympathetic vasoconstriction and increases the total peripheral resistance of the visceral vascular beds. Exposure to high +Gz has been shown to significantly decrease blood flow to the spleen, pancreas, liver, and kidneys, in an apparent effort to maintain blood flow to the brain and heart [4–6].

Fighter pilots are frequently and repeatedly subjected to high +Gz. Their blood and other body fluids flow along the direction of the inertial force to the lower body, and are redistributed in the body [3]. Previous studies have reported a possible association between impaired renal function and +Gz changes, but the reason for renal dysfunction observed in these pilots is unclear. The manner in which the hypergravity-induced hemodynamic changes decrease renal blood flow and cause renal hypoxia and the effects of hypergravity exposure on oxidative stress parameters are additional questions that need to be answered. Although investigations on the effects of exposure to hypergravity in various organs and tissues have been performed in a considerable number of animal and human studies, data regarding the effects of hypergravity exposure on the kidneys are scarce.

Acute or chronic kidney injury results from various insults and pathological conditions, and it is accompanied by the activation of compensatory repair mechanisms. Both insults and repair mechanisms are initiated by circulating factors, whose cellular effects are mediated by the activation of selective signal transduction pathways. The inducible nitric oxide synthase (iNOS) pathway is one of the signaling pathways that are activated during these processes. iNOS is one of the three isoforms of NOS, each of which differ in their localization, regulation and catalytic properties and are encoded by different genes [7]. iNOS is responsible for the synthesis of nitric oxide (NO) and is normally undetectable under physiological conditions, but can be expressed *de novo* under experimental or pathological conditions in a wide range of cells and tissues. When induced by various growth factors, cytokines, signaling pathways, or agents, iNOS continuously produces NO until the enzyme is degraded [8]. Mass production of NO and NO-derived substances is mainly ascribed to excessive induction of iNOS, and these products exert various effects on the kidneys. High production of NO by iNOS can have beneficial microbicidal, antiviral, antiparasitic, and antitumor effects [3, 8, 9], but aberrant iNOS induction may have detrimental consequences and seems to be involved in the pathophysiology of conditions such as tumor development, transplant rejection, and septic shock, in diseases such as asthma, rhinitis, multiple sclerosis, psoriasis, and neurodegenerative diseases [7, 9, 10]. The regulation of iNOS expression following exposure to hypergravity, a condition that can adversely affect the kidneys, has not yet been described. The purpose of this study was to examine whether exposure to hypergravity would induce renal hypoxia and would alter iNOS expression and NO production in the kidneys of mice.

## RESULTS

Upon gross inspection, the bilateral kidneys of all the mice appeared normal. No discoloration, hemorrhage, nodularity, shrinkage, or scarring was detected on the capsular surfaces. The centrifuged mice showed no significant alterations in the size or weight of their kidneys. All the parts of the kidneys could be cut with great ease. The renal cut surface of the centrifuged mice revealed no pathological abnormalities; no evidence of infarction, hemorrhage, mass or cystic lesion was identified. The pelvicalyceal system remained intact. Histopathologically, the centrifuged mice showed no significant morphological changes compared to those in the control group; no evidence of glomerular capillary wall lesion, mesangial hypercellularity, tubular atrophy, or interstitial fibrosis was detected. None of the centrifuged mice displayed significant morphological changes in renal vasculature.

**Figure 1:**
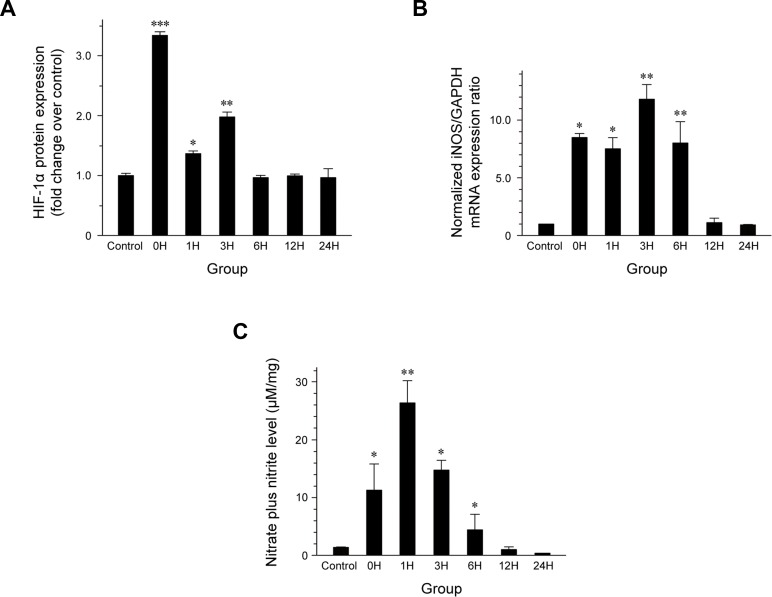
Effect of exposure to hypergravity on (A) renal HIF-1α expression, (B) iNOS mRNA expression, and (C) NO production **A.** Two peaks are observed at 0 and 3 h post-exposure. A 3.3-fold increase in HIF-1α protein is observed immediately after exposure (*P* < 0.001), and this increase persists until 3 h post-exposure (*P* = 0.008). **B.** iNOS mRNA expression is significantly elevated immediately after exposure to hypergravity (*P* = 0.022), and it increases further at 3 h post-exposure (*P* = 0.006). **C.** Effect of exposure to hypergravity on renal NO production. A nitrate/nitrite assay shows similar trends in the levels of iNOS expression, both of which are significantly increased from 0 to 6 h post-exposure. The nitrate/nitrite level reaches a peak at 1 h post-exposure. *P < 0.05; *P < 0.01; ***P < 0.001.

HIF-1α levels were significantly elevated immediately after exposure to hypergravity (0 h post-exposure, *P* < 0.001; Figure 1A), indicating that the exposure to hypergravity induced hypoxia in the kidneys of the centrifuged mice. In addition, the HIF-1α levels were significantly increased once more at 3 h post-exposure (*P* = 0.008), resulting in a double peak pattern. After a decrease at 6 h post-exposure, the HIF-1α protein levels were maintained at the level found in the control group until the end of the observation period.

Quantitative analysis of iNOS mRNA expression (Figure 1B) revealed a significant increase at 0 h post-exposure (*P* = 0.022), which persisted until 6 h post-exposure. The maximal expression levels were observed at 3 h post-exposure (*P* = 0.006), which resulted in a biphasic pattern. Throughout the remainder of the post-exposure period, the iNOS mRNA levels were maintained at the level found in the control group.

The nitrate/nitrite level showed an immediate obvious increased after exposure to hypergravity (*P* = 0.025) and remained significantly high until 6 h post-exposure, similar to the expression of iNOS mRNA (Figure 1C). It reached a peak at 1 h post-exposure (*P* = 0.005) and then began to decrease, until it reached a value even lower than that in the control group after 12 h post-exposure.

**Figure 2:**
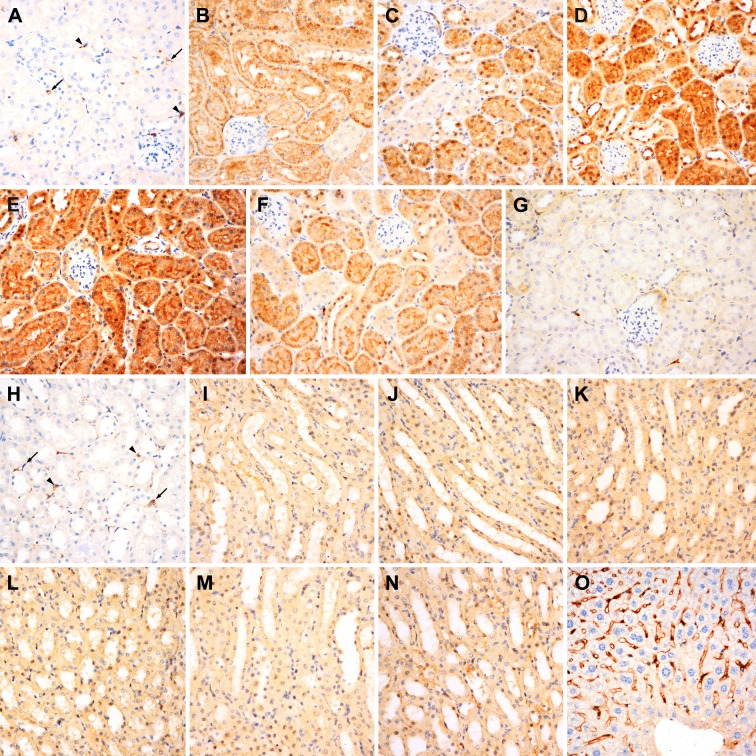
Immunostaining for iNOS in the renal cortex (A to G) and medulla (H to O) **A.** Renal cortex of the control mice. No iNOS expression is observed in the glomeruli or tubules in the cortex, whereas the peritubular capillaries (arrows) and inflammatory cells (arrowheads) located in the interstitium show weak to moderate iNOS immunoreactivity and serve as positive internal controls. (B to F) Renal cortex of the centrifuged mice. A significant time-dependent change in iNOS expression is noted in the renal tubular epithelial cells. Significantly increased levels of cytoplasmic iNOS expression persist between 0 and 12 h post-exposure. **B.** The 0-h interval group. iNOS expression is evident immediately after centrifugation. **C.** The 1-h interval group. **D.** The 3-h interval group. **E.** At 6 h post-exposure, maximum iNOS immunoreactivity is noted. **F.** The 12-h interval group. **G.** At 24 h post-exposure, iNOS expression disappears. **H.** Renal medulla of the control group. iNOS expression is absent in the medulla of the control kidney, except for the interstitial peritubular capillaries (arrows) and inflammatory cells (arrowheads) showing weak to moderate iNOS expression. **I.** to **N.** In contrast to the cortex, the medulla shows no significant time-dependent alteration in iNOS immunoreactivity. All the experimental groups shows weak cytoplasmic iNOS immunoreactivity in the tubular epithelium. **O.** Normal liver parenchyma is used as a positive control. There is a sharp demarcation of bile canaliculi by iNOS, whereas the cytoplasm of hepatocytes shows no iNOS expression.es shows no iNOS expression.

Figure 2 shows the immunostaining results of the cortical (Figure 2A-2G) and medullary (Figure 2H-2O) sections. The peritubular capillaries and some inflammatory cells located in the interstitium exhibited weak to moderate iNOS expression (Figure 2A). The control group did not display any iNOS immunoreactivity in the renal tubular structures and glomeruli, whereas the group exposed to hypergravity showed a significant time-dependent increase in iNOS protein expression in the cortex. A variable degree of cytoplasmic iNOS immunoreactivity was identified in the renal tubular epithelial cells of the centrifuged mice. The intensity of expression was moderate (0-, 1-, and 12-h interval groups) to strong (3- and 6-h interval groups), and the distribution was patchy (0-, 1-, 3-, and 12-h interval groups) to diffused (6-h interval group). Significantly increased levels of iNOS expression persisted in the cortical tubular epithelium between 0 and 12 h post-exposure (Figure 2B-2F). iNOS expression was evident immediately after centrifugation. At 6 h post-exposure, maximum iNOS immunoreactivity with diffused and strong expression was observed. The intensity and distribution pattern of iNOS in the 12-h interval group was nearly the same as that in the 0-h interval group. At 24 h post-exposure, there was no iNOS expression (Figure 2G).

iNOS expression was absent in the medulla of the kidneys of mice in the control group. Similar to the observations in the cortex, weak to moderate iNOS immunoreactivity was noted in the interstitial peritubular capillaries and inflammatory cells (Figure 2H). However, in contrast to the cortex, the medulla did not show any significant time-dependent alteration in iNOS expression. All the experimental groups showed weak cytoplasmic iNOS immunoreactivity in the tubular epithelium (Figure 2I-2N). Normal liver parenchyma was used as a positive control. There was a sharp demarcation of bile canaliculi by iNOS, whereas the cytoplasm of hepatocytes showed no iNOS expression (Figure 2O).

## DISCUSSION

The kidneys are particularly susceptible to hypoxic injury in many clinical conditions. One reason for renal sensitivity to hypoxia is that the renal microvasculature is highly complex and must meet a high energy demand. Therefore, factors related to renal hypoxia will considerably influence renal function and fluid balance. Direct damage and stress response caused by hypergravity can induce significant hemodynamic differences between the upper and lower body, in visceral organs and on the body surface. In this study, we investigated the effect of exposure to hypergravity on the expression of HIF-1α and iNOS and the production of NO in the kidneys of mice. We hypothesized that hypergravity exposure would induce renal hypoxia and affect the expression of HIF-1α and iNOS. In order to verify our hypothesis, we used a mouse model of short-term exposure to hypergravity and observed that the induction of HIF-1α was evident immediately after centrifugation, indicating that exposure to hypergravity induces renal hypoxia. Furthermore, the elevated HIF-1α levels were sustained for 3 h post-exposure. Distinct mechanisms are involved in the alteration in HIF-1α expression in hypoxic and normoxic conditions. Although they include multiple complex pathways and regulatory factors, in a hypoxic environment, HIF-1α levels are regulated mainly by protein degradation rather than by transcription [11]. However, in the normoxic state, various signaling pathways such as the mammalian target of rapamycin (mTOR) pathway and mitogen-activated protein kinase (MAPK) pathway activate or suppress the translation of HIF-1α [12–15]. Based on this, we speculate that the further increase in HIF-1α levels at 3 h post-exposure might be attributable to the activation of the mTOR and/or MAPK pathways rather than to oxygen-dependent regulation of HIF-1α degradation. However, further investigation is necessary to clarify the association between the above-mentioned signaling pathways and hypergravity-induced alteration in HIF-1α levels.

We also noted that significantly elevated iNOS mRNA levels persisted until 6 h post-hypergravity exposure, with the highest expression seen at the 3 h post-exposure. This result was consistent with the results of immunohistochemical staining of the tubular epithelial cells, which showed significantly increased cytoplasmic iNOS immunoreactivity immediately after hypergravity exposure and a sustained, strong intensity for 6 h. The sustained increase in HIF-1α levels in the centrifuged group compared to that in the control group during the reoxygenation period indicated that HIF-1α was involved in the increase of iNOS expression [16, 17]. Nitrate/nitrite levels showed a similar pattern to iNOS expression and they were maintained at an elevated state during the reoxygenation period, which suggests that iNOS increases NO production during hypoxia/reoxygenation injury caused by exposure to hypergravity. Subsequently, the increased NO level plays a role in hypoxia/reoxygenation injury. The role of NO in the kidney is complex: it can either attenuate or exacerbate renal injury, depending on the balance between its beneficial hemodynamic effects and its cytotoxic effects [18–20]. The site and rate of NO production, its chemical fate, and the differences in the temporal expression patterns of NOS determine this balance [21–24]. Interestingly, we observed that iNOS expression showed different patterns in the renal cortex and renal medulla. Blood flow and tissue oxygen tension are normally low in the renal medulla [25]. As a result, the renal medulla is known to be more sensitive to hypoxia than the renal cortex. While changes in iNOS expression in the renal medulla mildly increased immediately after exposure to hypergravity and no changes were observed during the reoxygenation period, iNOS immunoreactivity in the renal cortex showed moderate intensity immediately after exposure and strong intensity during the reoxygenation period. These findings can serve as evidence for the hypothesis that iNOS attenuates hypoxia/reoxygenation injury in the renal cortex.

In conclusion, we showed that exposure to hypergravity significantly increased the renal HIF-1α level immediately after exposure, and that a high HIF-1α level was maintained for 6 h post-exposure, raising the possibility that both hypoxia and reoxygenation may be involved in the upregulation of HIF-1α in the kidneys of mice exposed to hypergravity. In addition, we also demonstrated that exposure to hypergravity increased iNOS mRNA and protein expression immediately after exposure and during the reoxygenation period. The renal cortex of the centrifuged mice exhibited moderate to strong iNOS immunoreactivity in the tubular epithelial cells during the reoxygenation period, whereas in the renal medulla, iNOS immunoreactivity displayed only weak intensity, suggesting that iNOS expression induced by hypergravity exposure plays a protective role in the cortex against renal hypoxia/reoxygenation injury. Further investigation is necessary to clarify the role of iNOS and NO in kidneys exposed to hypergravity.

## MATERIALS AND METHODS

### Animals and hypergravity exposure

ICR mice at 7 weeks of age were purchased, fed standard laboratory mouse chow, provided with free access to water, and maintained on a 12-h light-dark cycle under temperature- and moisture-controlled conditions (20-25°C and 40-45%, respectively). In order to avoid the effects of unfavorable factors that include fear and stress, the mice were allowed to acclimatize to the rearing environment for 7 d. They were deprived of food for 12 h before the beginning of each experiment. The mice were then exposed to short-term hypergravity at +3Gz for 1 h (onset rate, +0.5Gz/s to +1Gz/s) using a small-animal centrifuge. Each mouse was placed into a cylindrical plastic restraint device that, when mounted in a centrifuge, allowed +Gz to be delivered along the rostro-caudal axis. After the mice were secured, the restraint device was clamped to the end of the centrifuge arm, which allows one degree of freedom, thereby ensuring that the net G field was perpendicular to the floor of the restraint device. The mice in the control group (*n* = 12) were placed in the centrifuge arm and underwent a similar process to the one described above, but they were not exposed to hypergravity. The behavior of the mice was monitored with a CCD camera throughout the centrifugation experiments. The Institutional Animal Care and Use Committee of the Republic of Korea Air Force Aerospace Medical Center approved all experimental procedures involving the animals

To investigate the time course of iNOS mRNA expression, hypoxia-inducible factor (HIF)-1α expression, NO production, and iNOS protein expression, the centrifuged mice were randomly divided into six groups. At 0 h (*n* = 9), 1 h (*n* = 9), 3 h (*n* = 9), 6 h (*n* = 9), 12 h (*n* = 9), and 24 h (*n* = 9) after the cessation of centrifugation, the mice were euthanized by cervical dislocation and laparotomized via midline incision. The kidneys were removed from the animals and immediately preserved in 10% buffered formalin solution or frozen in liquid nitrogen and stored at −70°C for further analyses.

### Quantitative real-time reverse-transcriptase polymerase chain reaction (RT-PCR)

iNOS mRNA expression was detected in the centrifuged mice and compared with that in the control mice. Total kidney RNA was isolated using the NucleoSpin RNA II extraction kit (Macherey-Nagel GmbH & Co. KG, Dueren, Germany). A ReverTra Ace-α- reverse-transcriptase kit (Toyobo Co., Ltd., Osaka, Japan) was used for cDNA synthesis. The amount of standard cDNA was determined photometrically. The reverse-transcribed cDNA was subjected to real-time RT-PCR using SsoAdvanced SYBR Green Supermix (Bio-Rad Laboratories, Inc., Hercules, CA, USA). PCR was performed using the Bio-Rad CFX96 Real-Time PCR Detection System with a C1000 Thermal Cycler (Bio-Rad Laboratories, Inc.). The primer sequences used for iNOS were as follows: forward 5′-GGAGCGAGTTGTGGATTG-3′; reverse 5′-CCAGGAAGTAGGTGAGGG-3′. The primer sequences used for GAPDH were as follows: forward 5′-CAAGAAGGTGGTGAAGCA-3′; reverse 5′-GGTGGAAGAGTGGGAGTT-3′. The PCR for iNOS and GAPDH was initiated with a denaturing step at 95°C for 3 min, followed by 40 cycles at 95°C for 10 s, 58°C for 10 s, and 72°C for 20 s. A melting curve, ramping from 65°C to 95°C, was performed following each RT-PCR to test for the presence of primer dimers. When primer dimer formation was detected, the PCR was rerun using a separate aliquot of cDNA. Each measurement was repeated three times, and the values were used to calculate the ratio of iNOS to GAPDH, with the control set to a value of 1.0 as a calibrator. The normalized expression ratio was calculated using the 2^−ΔΔCt^ method [26].

### Histopathology and histochemistry

Formalin-fixed kidney tissues were sectioned, paraffin-embedded, and processed for routine histology staining, including hematoxylin-eosin, periodic acid-Schiff, Masson trichrome, and periodic acid methenamine-silver, and immunohistochemical staining.

### Immunohistochemistry

Immunohistochemical staining for iNOS was performed using the Bond Polymer Intense Detection System (Vision Biosystems, Mount Waverley, Victoria, Australia). In brief, 4-μm thick sections of formalin-fixed, paraffin-embedded kidney tissues were deparaffinized using Bond Dewax Solution (Vision BioSystems), and an antigen retrieval procedure was performed using Bond ER Solution (Vision BioSystems) for 30 min at 100°C. Endogenous peroxidases were quenched by incubation with hydrogen peroxide for 5 min. The sections were incubated for 15 min at ambient temperature with a rabbit polyclonal anti-iNOS antibody (1:100; Abcam, Cambridge, MA, USA). The biotin-free polymeric horseradish peroxidase-linker antibody-conjugate system was used in the Bond-maX automatic slide stainer (Vision BioSystems), and visualization was performed using a 3,3-diaminobenzidine (DAB) solution (1 mM DAB, 50 mM Tris-HCl buffer [pH 7.^6^], and 0.006% H_2_O_2_). Nuclei were counterstained with hematoxylin. The slides were subsequently dehydrated following a standard procedure and sealed with coverslips. To minimize inter-assay variation, positive and negative control samples were included in each run. The positive control sample was a healthy liver tissue. The negative control was prepared by substituting non-immune serum for the primary antibody. No detectable staining was evident.

### Nitrate/nitrite measurement

Tissue samples were ground into a fine powder in liquid nitrogen and homogenized in a solution containing 10 mM 4-(2-hydroxyethyl)-1-piperazineethanesulfonic acid (pH 7.2), 0.32 M sucrose, 0.1 mM ethylenediaminetetraacetic acid, 1 mM dithiothreitol, several protease inhibitors (10 μg/mL each of leupeptin, pepstatin A, chymostatin, antipain, and soybean trypsin inhibitor), and 100 μg/mL phenylmethylsulfonyl fluoride. NO production was indirectly quantified by measuring the nitrate/nitrite levels in the supernatants from the kidney tissue homogenates using a Nitrate/Nitrite Colorimetric Assay Kit (Cayman Chemicals, Ann Arbor, MI, USA), following the manufacturer's instructions. Briefly, 5-μL aliquots were injected into the Sievers Nitric Oxide Analyzer (NOA 280i; Sievers Instruments, Boulder, CO, USA) and pelleted by centrifugation using acetic acid as a reductant for nitrite, and vanadium chloride and hydrogen chloride as reductants for nitrate and sodium iodide, respectively. The results were normalized for protein concentration as assessed using the Bradford reagent. The nitrate/nitrite concentration was expressed as μM based on tissue weight.

### Enzyme-linked immunosorbent assay

Preparation of lysates from tissue samples was performed as described above. The protein concentrations of HIF-1α were determined using a commercially available enzyme-linked immunosorbent assay kit (R&D Systems, Inc., Minneapolis, MN, USA).

### Statistical analysis

All the values are reported as mean ± standard error. The differences in the normalized mRNA ratio, protein concentration, and nitrite/nitrate concentration between the groups were assessed using the Student's *t*-test (Statistical Package for the Social Sciences [SPSS] version 18.0 software; IBM SPSS, Chicago, IL, USA), and P values less than 0.05 were considered statistically significant.
